# Spontaneous coronary artery dissection in the context of tamoxifen; Is there any correlation?

**DOI:** 10.1002/ccr3.9140

**Published:** 2024-07-05

**Authors:** Homina Saffar, Leili Abdan, Zahra Abdan, Hamidreza Hekmat, Alireza Amirzadegan, Negar Omidi

**Affiliations:** ^1^ Student Research Committee, Faculty of Medicine Mazandaran University of Medical Sciences Sari Iran; ^2^ Cardiovascular Disease Research Institute, Tehran Heart Center, School of Medicine, Tehran University of Medical Sciences Tehran Iran; ^3^ Clinical Research Development Center Imam Reza Hospital, Kermanshah University of Medical Sciences Kermanshah Iran; ^4^ School of Medicine, baharloo Hospital, International Campus, Tehran University of Medical Sciences Tehran Iran; ^5^ Tehran Heart Center, Cardiovascular Diseases Research Institute, Tehran University of Medical Science Tehran Iran; ^6^ Cardiac Primary Prevention Research Center, Cardiovascular Diseases Research Institute, Tehran University of Medical Science Tehran Iran

**Keywords:** acute coronary syndrome, breast cancer, chest pain, myocardial infarction, spontaneous coronary artery dissection, tamoxifen

## Abstract

**Key Clinical Message:**

Clinicians should consider spontaneous coronary artery dissection in middle‐aged women presenting with acute coronary syndromes and a history of tamoxifen use, to ensure timely diagnosis, and appropriate management strategies.

**Abstract:**

Spontaneous coronary artery dissection (SCAD) is characterized by a non‐iatrogenic, nontraumatic separation of the coronary artery wall, contributing to acute coronary syndromes (ACS), and sudden cardiac death. SCAD predominantly affects the left anterior descending artery (LAD) and is frequently observed in middle‐aged women. This condition has been associated with cancer treatment and exogenous hormones exposure. The diagnostic gold standard remains coronary angiography, management strategies include conservative measures, percutaneous coronary intervention (PCI), and coronary artery bypass graft surgery (CABG). We describe a case of a 54‐year‐old woman with breast cancer and a history of tamoxifen use, presenting with SCAD in the posterolateral branch (PLB) originating from the left circumflex artery (LCX), and right coronary artery (RCA) and managed conservatively.

## 1 INTRODUCTION

Spontaneous coronary artery dissection (SCAD) represents a rare etiology of acute coronary syndromes (ACS), characterized by non‐iatrogenic, nontraumatic separation of the coronary artery wall.^1^, ^2^ SCAD can affect any of the coronary arteries, with the left anterior descending artery (LAD) being the most frequently involved, followed by the right coronary artery (RCA), and rarely the left main coronary artery (LMCA). This occurs due to spontaneous, non‐atherosclerotic separation of the coronary artery wall, resulting in an intramural hematoma (IMH) in the false lumen, which may also be associated with an intimal tear.^3^, ^4^, ^5^ Studies have estimated that SCAD accounts for 0.1%–0.4% of ACS cases, which has increased to 4% in recent years. Additionally, SCAD has been identified as the cause of 0.5% of sudden cardiac death cases after autopsy.^6^, ^7^

Clinical symptoms of SCAD are typically similar to those of atherosclerotic ACS and may include chest pain or equivalent symptoms, ECG changes, and elevated cardiac biomarkers. In some cases, it can lead to heart failure (HF), ventricular arrhythmias, and sudden cardiac death. The typical patient phenotype is a middle‐aged woman without traditional cardiac risk factors.^3^, ^8^ Predisposing factors may include genetics, hormonal fluctuations, pregnancy, systemic inflammatory disease, and arteriopathy. Known triggers of the disease include physical activity, particularly in men, and emotional stress, especially in women. Cancer treatment and exposure to sympathomimetic drugs are also associated with SCAD.^1^, ^3^, ^9^ While coronary angiography serves as the gold standard and primary imaging modality for diagnosing patients presenting with ACS, additional imaging techniques such as optical coherence tomography (OCT), intravascular ultrasound (IVUS), and cardiac magnetic resonance (CMR) may also be utilized.^6^, ^8^

SCAD treatment methods include conservative management, percutaneous coronary intervention (PCI), and coronary artery bypass graft surgery (CABG). The mainstay of medical therapy for SCAD is beta‐blockers. Administration of angiotensin‐converting enzyme inhibitors (ACEIs), angiotensin receptor blockers (ARBs), or mineralocorticoid receptor antagonists is recommended, particularly in cases of HF. There is controversy in the use of antiplatelets and anticoagulants. The use of thrombolytic drugs is not recommended due to the possibility of extension of dissection or hematoma.^3^, ^7^, ^10^ Given the lack of awareness about this condition among health care providers, as well as the current treatment challenges, we decided to report a case of SCAD.

## 2 CASE PRESENTATION

A 54‐year‐old female, with underlying breast cancer, presented to the emergency department (ED) with chest pain that had started 8 h prior to admission, described as retrosternal with radiation to the left arm, and characterized as tightness and heaviness. The patient had no prior history of similar episodes and reported that the pain began while driving. At the onset, the pain was most intense but gradually diminished, persisting albeit at a reduced intensity. Notably, the patient did not have a history of hypertension, diabetes, dyslipidemia, smoking, or familial heart disease and did not have a recent delivery. Due to underlying breast cancer, the patient had previously undergone surgical resection, as well as chemotherapy and radiotherapy. The patient reported taking tamoxifen and zoledronic acid every 6 months. The patient's vital signs were within normal range, and physical examination was unremarkable.

## 3 DIAGNOSTIC FINDINGS

**FIGURE 1:**
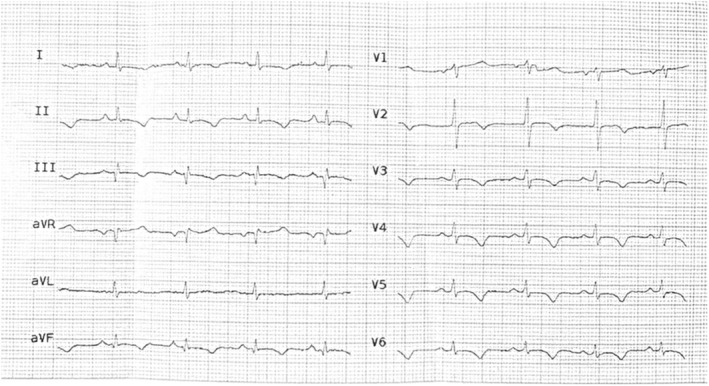
Twelve lead electrocardiogram upon admission demonstrates inverted T waves in precordial, lateral and inferior leads and ST coving in V1–V5.

In the ED, the initial ECG showed inverted T waves in precordial, lateral, and inferior leads and ST coving in V1–5. (Figure 1) Laboratory tests were normal except for elevated serum troponin, measured at 19224 μg/L. Transthoracic echocardiogram (TTE) revealed moderate systolic dysfunction (LVEF = 35%) and akinesia of left ventricular (LV) apical segment, mid anterior, and mid lateral wall. The patient received treatment with aspirin, clopidogrel, metoprolol, atorvastatin, and nitroglycerin, with her troponin level subsequently rising to 29,215 μg/L.

**FIGURE 2:**
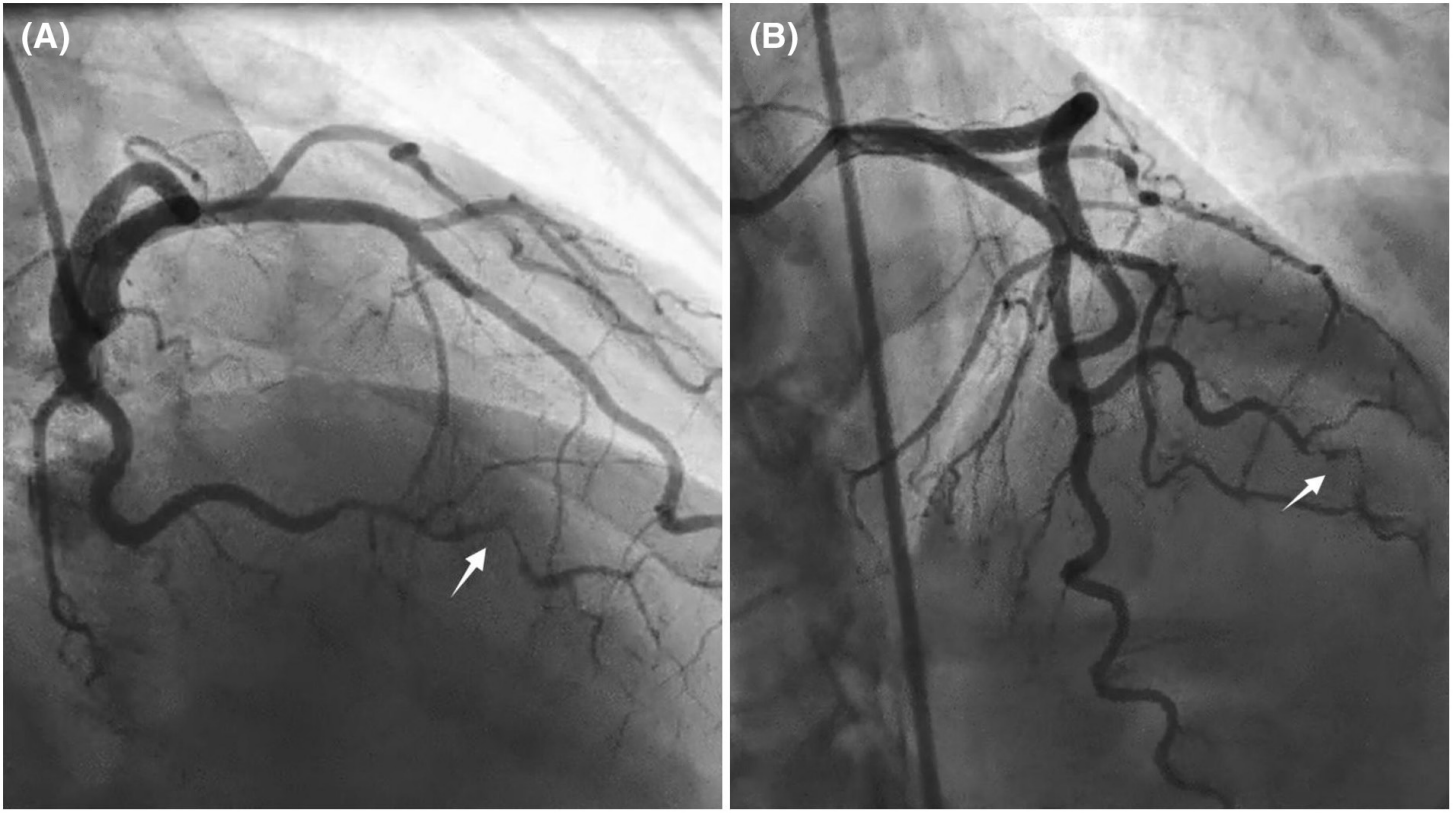
Angiography images showing a dissection at the proximal portion of posterolateral branch originating from left circumflex artery (white arrow) in (A). RAO (right anterior oblique) caudal and (B). LAO (left anterior oblique) cranial view.

**FIGURE 3:**
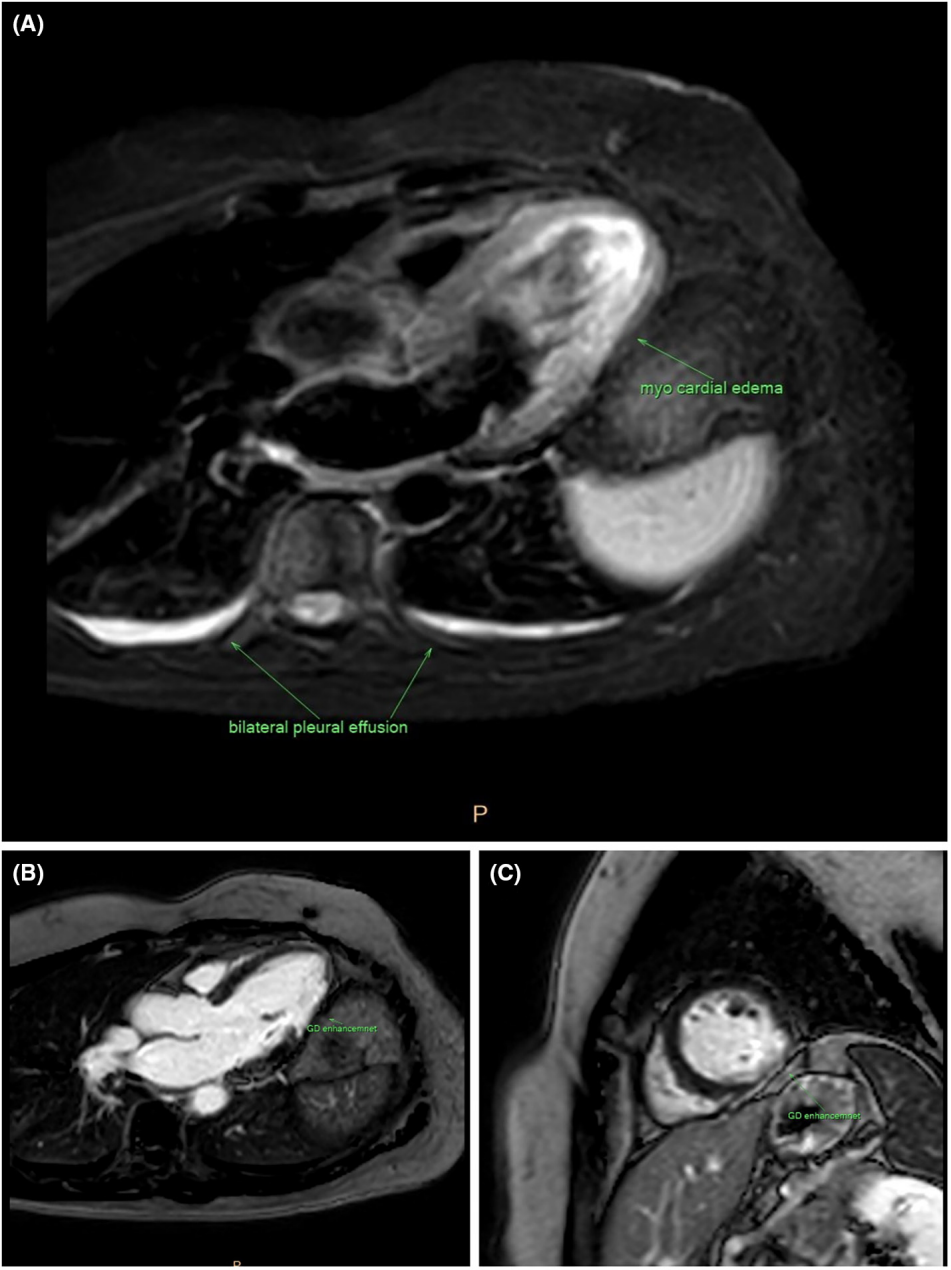
Cardiac magnetic resonance (CMR) imaging of the patient represent: (A) T2 weighted sequence (STIR) reveals increased myocardial signal intensity in mid to apical inferolateral segment of LV with bilateral mild pleural effusion. (B) Late gadolinium sequence in short axis view demonstrates sub‐endocardial to mid wall (nearly transmural) gadolinium enhancement in mid to apical inferoposterior segment of LV. (C) Late gadolinium enhancement in three‐chamber view reveals sub‐endocardial to mid wall (nearly transmural) gadolinium enhancement in mid to apical inferoposterior segment of LV.

Based on the ECG alterations, increased serum troponin, and decreased EF, coronary angiography was performed, revealing a posterolateral branch (PLB) lesion suggesting SCAD. (Figure 2) To confirm the diagnosis, CMR imaging was performed, displaying an acute ischemic infarct in the mid to apical inferolateral wall, along with significant regional wall motion abnormalities (RWMA) and edema in the mid to apical segments. Additionally, severe reduction in systolic function (LVEF = 30%) and subendocardial to transmural late gadolinium enhancement (LGE) in the mid to apical inferolateral wall were evident. (Figure 3).

## 4 TREATMENT AND FOLLOW‐UP

The patient was discharged with conservative treatment including aspirin, clopidogrel, metoprolol, atorvastatin, losartan, and empagliflozin. The patient remained asymptomatic during follow‐up visits. A follow‐up TTE 5 months later revealed improvement in LV systolic function (LVEF = 40%–45%), and the patient reported enhanced quality of life.

## 5 DISCUSSION

SCAD was previously a rare cause of ACS, but in recent years, its incidence has increased to 4% of ACS cases due to increased awareness among physicians. Presently, clear management guidelines for SCAD treatment are lacking, primarily relying on retrospective data and case series. In over two‐thirds of cases, the LAD is involved, with LAD involvement being more common in women and RCA involvement more prevalent in men.^4^, ^11^ Significantly, our patient was identified as codominant based on angiographic assessments, with SCAD occurring in the PLB stemming from the left circumflex artery (LCX) and RCA, a rare occurrence among SCAD cases. In a similar context, Valencia et al documented a case involving a 47‐year‐old woman with SCAD situated in the LCX and ramus, aligning with our findings.^12^ This finding may account for the decrease in the patient's ejection fraction (EF) based on the affected coronary territory.

The primary mechanism underlying SCAD remains unknown, but predisposing factors include genetics (such as fibromuscular dysplasia, Ehlers‐Danlos syndrome, and Marfan syndrome), exogenous hormones (such as oral contraceptives, infertility treatment, and postmenopausal hormone therapy), pregnancy, systemic inflammatory disease, and arteriopathy. Known triggers for SCAD include extreme physical or emotional stress, sympathomimetic drugs (such as cocaine and amphetamines), cancer treatment, and activities that increase thoracoabdominal pressure.^1^, ^2^, ^3^, ^9^ Our patient reported the use of tamoxifen due to an underlying history of breast cancer. Comanescu et al. reported a case of spontaneous carotid artery dissection in a 39‐year‐old female with a history of breast cancer who had undergone surgery, chemotherapy, radiotherapy, and a 3.5‐year course of tamoxifen. The patient presented to the ED with neurological symptoms, including seizures and altered consciousness. Magnetic resonance angiography (MRA) confirmed carotid dissection, and over a 12‐month follow‐up period, the patient's symptoms showed improvement.^13^ Hirsch et al. documented a case of SCAD in a 37‐year‐old transgender individual who had undergone an orchiectomy and was on gender‐affirming estrogen therapy. The patient presented with sudden chest pain and a rapid increase in troponin levels. Angiography revealed SCAD, with estrogen supplementation identified as a significant risk factor in this case.^14^ Furthermore, cases of SCAD occurring following hormonal fluctuations induced by oral contraceptives, topical hormone replacement therapy, and pseudomenopause therapy have been reported in the literature.^15^, ^16^ This association hints at a potential relationship between arterial dissection, and hormonal alteration following tamoxifen consumption. Long‐term tamoxifen administration leads to increased levels of circulating estrogens through disruption of normal negative pituitary feedback mechanisms and direct effects on granulosa cells.^17^, ^18^, ^19^ Notably, tamoxifen acts as a potent stimulator of ovarian function, inducing hyperestrogenism, with up to threefold rise in estradiol, estrone, and progesterone levels observed in premenopausal women treated with tamoxifen as a single agent.^18^, ^19^ The precise mechanism linking hormonal alterations to the onset of SCAD remains incompletely understood. However, it appears that tamoxifen, through its effects on estrogen receptors (ERα, ERβ, GPER), heparanase (HPSE), extracellular matrix proteases (MMPs), angiotensin‐II receptor type 1 (AT1R), and endothelin‐1 receptor type A (ETAR) at the cellular level, induces secondary alterations in the structure and integrity of the vascular endothelium. This includes changes in the balance of elastic fiber and mucopolysaccharide content, smooth muscle hypertrophy/hyperplasia, and mucinous cystic necrosis, all of which contribute to the development of SCAD.^16^, ^20^, ^21^, ^22^, ^23^, ^24^, ^25^

Clinical presentations of SCAD often mimic atherosclerotic ACS. The typical SCAD patient is a middle‐aged woman with no classic cardiovascular risk factors, with women accounting for 87%–95% of SCAD cases, and the average age at diagnosis is between 44 and 53 years old.^7^, ^9^ The degree of occlusion is typically related to the severity of disease symptoms, which can manifest as chest pain or equivalent symptoms, along with ST‐segment elevation or non–ST segment elevation alterations in the ECG and an increase in cardiac biomarkers. In less common cases, symptoms may manifest as ventricular arrhythmias (3%–10%), cardiogenic shock (<3%), or sudden cardiac death (<1%). Additionally, 44%–49% of patients experience a reduction in LV ejection.^3^, ^6^ In our case, the clinical manifestations comprised chest pain alongside ECG changes, elevated cardiac biomarkers, and subsequent HF. During the patient's angiography, the PLB was discovered to be separate from the LCX and RCA, indicating codominant coronary artery circulation in the patient, which could explain the decrease in EF.

The gold standard diagnostic modality for ACS patients is coronary angiography. SCAD is classified into three types based on angiographic images. Type 1, pathognomonic of SCAD, appears in a small number of patients, exhibiting several radiolucent lumens with extraluminal contrast staining. Type 2, the most common type, manifests as diffuse, smooth stenoses of varying severity and length (typically >20 mm). In comparison Type 3 represents focal narrowing (<20 mm in length) and is frequently misdiagnosed as atherosclerotic pathology. Given the limitations of coronary angiography, particularly in the diagnosis of Type 2 and Type 3 cases, supplementary imaging techniques are often beneficial.^6^, ^11^ Intracoronary imaging modalities (OCT and IVUS) offer more accurate assessments of arterial wall layers, although this requires instrumentation of the coronary artery—potentially worsening the condition—and is costly and less accessible. CMR serves as an additional imaging method aiding in confirming the diagnosis of SCAD by revealing delayed gadolinium enhancement in an area with suspected dissection. Notably, a normal CMR does not entirely exclude SCAD.^7^, ^8^, ^10^ Considering the lack of access to intracoronary imaging modalities, CMR was performed in the present case to confirm the diagnosis, revealing subendocardial to transmural late gadolinium enhancement in the mild to apical inferolateral wall. Given that clinical consequences of SCAD‐related noncoronary arteriopathies are uncommon and complications from these extracoronary arterial lesions in the short to medium term are extremely rare, an additional imaging modality for systematic arterial screening was not performed in this patient.^26^

The optimal management approach for patients with SCAD is not well established, with available treatment options including conservative strategies, PCI, or CABG. The primary goal of treatment during the acute phase is to maintain myocardial perfusion and cardiac function.^6^ Revascularization procedures are reserved for patients with ongoing ischemia, hemodynamic instability, refractory arrhythmia, and left main coronary dissection.^3^, ^4^ Medical treatment is preferred as the dissected coronary tends to improve spontaneously in 97% of cases.^2^, ^11^ Beta blockers are the cornerstone of SCAD treatment, as they reduce shear stress on the coronary artery walls and subsequently mitigate the risk of propagation and recurrence.^6^, ^11^ There is controversy regarding the initiation and duration of dual antiplatelet therapy (DAPT), although specific studies propose potential benefits from using P2Y12 inhibitors for 12 months.^4^, ^10^, ^11^ Other medications used to lower blood pressure and protect the myocardium, particularly in cases of HF, include ACEIs, ARBs, or mineralocorticoid receptor antagonists.^6^, ^7^ Anticoagulant treatment should be discontinued after confirming the diagnosis of SCAD, and its administration is recommended only in cases of revascularization, LV thrombus, or thromboembolism.^3^, ^4^, ^7^ The use of antithrombotic agents in the context of SCAD is contraindicated due to the potential for coronary rupture and subsequent cardiac tamponade.^4^, ^6^ The role of lipid‐lowering therapies in managing SCAD is unclear due to the lack of atherosclerosis involvement in its pathogenesis, but it is recommended for patients with underlying hyperlipidemia.^7^, ^11^ In this case, the patient was administered metoprolol, losartan, aspirin, clopidogrel, atorvastatin, and empagliflozin due to the absence of indications for revascularization therapy. Empagliflozin, a sodium‐glucose co‐transporter 2 (SGLT‐2) inhibitor, was utilized as a standard treatment for HF with reduced EF.^2^, ^27^

## 6 CONCLUSION

While the diagnosis and management of SCAD are critical for improving patient outcomes, there is a pressing need for increased awareness among clinicians. The potential link between tamoxifen and SCAD requires further investigation through targeted research initiatives. Large‐scale observational studies and mechanistic research focusing on the impact of hormonal alterations on vascular integrity should be prioritized to enhance our understanding of this complex relationship and develop strategies to mitigate the risk of SCAD. Clinicians must be vigilant for SCAD in middle‐aged women with a history of cancer treatment and hormone exposure, as early detection and appropriate management can lead to better long‐term outcomes. Conservative pharmacological treatment has demonstrated promise in improving cardiac function and quality of life in SCAD patients, particularly those with HF. By addressing these critical knowledge gaps and emphasizing proactive clinical approaches, we can improve the care and outcomes of SCAD patients.

## AUTHOR CONTRIBUTIONS

**Homina Saffar:** Writing – original draft; writing – review and editing. **Leili Abdan:** Writing – review and editing. **Zahra Abdan:** Data curation. **Hamidreza Hekmat:** Conceptualization; data curation. **Alireza Amirzadegan:** Conceptualization; data curation. **Negar Omidi:** Conceptualization; data curation; supervision.

## FUNDING INFORMATION

This research did not receive any grant from funding agencies in the public, commercial, or nonprofit sectors.

## CONFLICT OF INTEREST STATEMENT

Authors have no conflict of interests.

## ETHICS STATEMENT

There were no ethical considerations to be considered in this research.

## CONSENT

The written informed consent for publication was obtained from the patient.

## Data Availability

The data of this article will be shared on request.
